# Free-electron interactions with van der Waals heterostructures: a source of focused X-ray radiation

**DOI:** 10.1038/s41377-023-01141-2

**Published:** 2023-06-16

**Authors:** Xihang Shi, Yaniv Kurman, Michael Shentcis, Liang Jie Wong, F. Javier García de Abajo, Ido Kaminer

**Affiliations:** 1 Solid State Institute and Faculty of Electrical and Computer Engineering, Technion – Israel Institute of Technology, Haifa, 32000 Israel; 2 School of Electrical and Electronic Engineering, Nanyang Technological University, Singapore, 639798 Singapore; 3 ICFO–Institut de Ciencies Fotoniques, The Barcelona Institute of Science and Technology, Castelldefels, 08860 Spain; 4 ICREA–Institució Catalana de Recerca i Estudis Avançats, Passeig Lluís Companys 23, Barcelona, 08010 Spain

**Keywords:** X-rays, Metamaterials, Nanophotonics and plasmonics

## Abstract

The science and technology of X-ray optics have come far, enabling the focusing of X-rays for applications in high-resolution X-ray spectroscopy, imaging, and irradiation. In spite of this, many forms of tailoring waves that had substantial impact on applications in the optical regime have remained out of reach in the X-ray regime. This disparity fundamentally arises from the tendency of refractive indices of all materials to approach unity at high frequencies, making X-ray-optical components such as lenses and mirrors much harder to create and often less efficient. Here, we propose a new concept for X-ray focusing based on inducing a curved wavefront into the X-ray generation process, resulting in the intrinsic focusing of X-ray waves. This concept can be seen as effectively integrating the optics to be part of the emission mechanism, thus bypassing the efficiency limits imposed by X-ray optical components, enabling the creation of nanobeams with nanoscale focal spot sizes and micrometer-scale focal lengths. Specifically, we implement this concept by designing aperiodic vdW heterostructures that shape X-rays when driven by free electrons. The parameters of the focused hotspot, such as lateral size and focal depth, are tunable as a function of an interlayer spacing chirp and electron energy. Looking forward, ongoing advances in the creation of many-layer vdW heterostructures open unprecedented horizons of focusing and arbitrary shaping of X-ray nanobeams.

## Abstract (web summary)

X-ray self-focusing sources are demonstrated by free electrons coherently interacting with van der Waals heterostructures, bypassing the need for X-ray optics that are often inefficient and challenging to fabricate.

## Graphical abstract

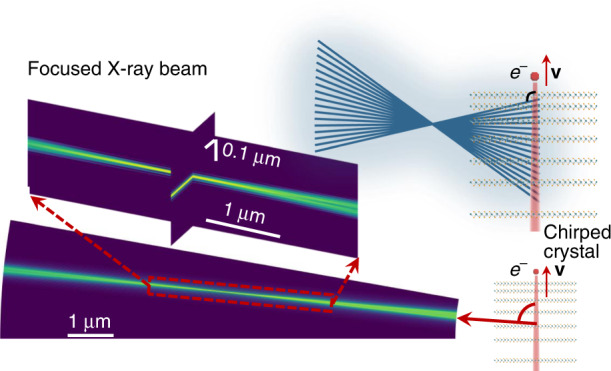

## Introduction

X-ray-based technology enables a wealth of applications in fundamental science^1–4^, medical imaging^5^, security scanners, industrial quality control, and many more fields^6^. However, the intrinsically weak interaction between X-rays and matter limits the ability to coherently manipulate X-ray waves using optical components. This limit is especially pronounced when compared to the abundance of methods of coherent wave shaping in the optical regime, from high-quality lenses to phase masks and spatial light modulators^7–9^. Such optical elements opened the way to breakthroughs and important applications in the optical regime^10–12^. It is a long-standing challenge to transfer more novel ideas that rely on coherent wave shaping from the optics to X-ray science^13^.

Certain coherent manipulations of X-ray waves are accessible using state-of-the-art zone-plates^1,14^ and Bragg mirrors^15,16^. However, such manipulations often necessitate high-quality X-ray beams that are only available in large facilities such as synchrotrons and free-electron lasers^17^. These facts prevent the wider spread of X-ray applications to more compact platforms, especially the applications that benefit from coherent wave shaping^2,3,18^.

The focusing of X-ray waves on nanoscale spot sizes and microscale focal distances is especially challenging. Using reflective optics for such purposes is usually limited by the quality of the mirror surface, which generally requires nanometer to sub-nanometer roughness over tens of micron apertures^19^. In refractive and diffractive optics, a large numerical aperture (NA) and short focal length can be achieved by compound devices, but typically at the cost of loss of coherence and low efficiency^20^.

Here, we propose a different strategy, integrating the focusing operation to be part of the X-ray generation mechanism. We rely on recent breakthroughs in two-dimensional (2D) materials that can be engineered on the atomic scale and show how to utilize such materials for focusing of the emitted radiation with diffraction-limited^21–24^ hotspots during the X-ray generation process. In particular, van der Waals (vdW) materials have been shown useful for tunable X-ray generation^25–27^. This X-ray generation process is based on the interaction of free electrons with the crystalline structure of the material, in a process known as parametric X-ray (PXR) radiation. Our work now shows how engineering many-layers vdW heterostructures can alter the intrinsic interaction of the electrons with the crystalline structure in a way that alters the phase-front of the emitted wave, producing coherently shaped X-rays.

Our work uses the unique properties of vdW heterostructures to integrate the X-ray wave shaping into the emission process, creating a unified source that produces shaped X-ray wavepackets. The efficiency remains equivalent to that of the original free-electron-based source, bypassing the losses from X-ray optics. Specifically, we implement this concept by carefully designing aperiodic vdW heterostructures with focusing parameters that can be tuned by customizing the chirp of the crystal periodicity. We demonstrate our scheme by a full-wave numerical simulation and compare the radiation to that of a conventional periodic vdW material. As an example, we present a focusing X-ray beam at 4 keV photon energy with a diffraction-limited beam width of just ~10 nm, at a focal distance of 10 μm.

Looking at the bigger picture, research on vdW materials and their heterostructures has opened new avenues to access versatile material properties. Custom-designed vdW heterostructures have revealed exotic phenomena and novel applications that are not accessible by the constituent layers alone, such as 2D superconductivity^28^, atomic-scale transistors and diodes, quantum capacitance, and tunneling devices^29,30^. Nevertheless, the prospects of such custom-built heterostructures in X-ray science have so far remained unexplored.

Of special importance to our work is the tunability of the interlayer spacings in vdW materials, which can be tuned in reversible ways via intercalation^31–34^, pressure^35,36^, temperature^37^, and optical excitation^38,39^. As an example, tuning the interlayer spacing of MoS_2_ has found important applications in energy storage, catalysis, and environmental remediation^32^. In intercalation, the addition of foreign species, such as polyethylene oxide (PEO), to MoS_2_ during the exfoliation/restacking steps can expand the interlayer spacing, with the extension controlled by the species and the infiltration densities^31,32^. Therefore, one can create a chirped vdW heterostructure by intercalating different foreign species accompanied by different infiltration densities across the layers.

This tunability in interlayer spacings is precisely the degree of freedom that we use below. It is noteworthy that our proposed methods for generating shaped X-rays from crystalline materials are not limited to vdW structures. Traditional processes, such as atomic layer deposition, can grow layered atomic-thick crystalline films with precise thickness control^40^. However, conventional crystals have strong bonds across layers and, thus, are more limited in the choice of materials that can be grown on top of one another. A well-known limitation is a requirement for transverse lattice-matching^29,41–44^, which could cause dislocation or strain when not perfectly matched. In contrast, the layered structure of vdW materials allows different materials to be bonded via the relatively weak vdW forces, which reduces the challenge of tuning interlayer spacing. The large number of vdW materials that are currently available allows a wide range of combinations compared with traditional methods for crystal growth. Part of this versatility arises from the weak interlayer bonds relative to the strong intralayer bonds of van der Waals (vdW) materials, bypassing traditional constraints of lattice-matching^29,41–44^.

The techniques for the vertical assembly of different vdW materials usually boil down to the construction of heterostructures one monolayer at a time with mechanical and deposition-based methods^30^. Large-scale assembly techniques^44–46^ have been implemented for scalable and practical manufacturing of vdW heterostructures. With rapid advances in integration technologies, vdW heterostructures have improved from a few to tens of layers^47^, reaching micrometer-scale nanowire heterostructures^48,49^. Hybrid vdW and conventional bulk-material heterostructures^50^ bring more flexibility in fabricating bulky crystalline structures. In our work below, we consider both the traditional structures made layer-by-layer, and thicker structures made by stacking multiple slabs of nanoscale thicknesses, each composed of tens to hundreds of layers.

Our proposal in this work is inspired by analogous schemes for beam shaping and focusing that were investigated in the optical regime using chirped gratings^51,52^ for a modified Smith–Purcell radiation^53–55^. A recent experiment reported the first observation of this effect using a chirped grating for optical Smith–Purcell-type radiation inside a scanning electron microscope^52^. Our study can be seen as a complementary scheme wherein the grating is realized on the atomic scale using a versatile crystalline material. Specifically, vdW heterostructures provide this versatility, allowing us to realize beam shaping and diffraction-limited focusing in the X-ray regime. Importantly, we highlight a fundamental difference between our heterostructure approach and the Smith–Purcell-type approach—a difference that goes beyond the choice of wavelength: whereas Smith–Purcell-type effects all rely on electrons passing by a grating, grazing the surface at a certain distance, the PXR-type effects that we study here rely on electrons penetrating the material, interacting with its bulk. The interaction with the bulk material triggers emission from multiple atomic layers that must coherently interfere for an effective emission process. This additional condition, when properly satisfied, can provide higher monochromaticity and directionality for the emitted radiation^26^.

## Results

**Fig. 1:**
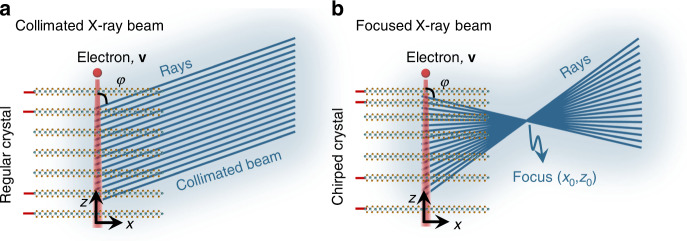
X-ray beam focusing created by free-electron interaction with a custom-made van der Waals (vdW) heterostructure. This illustration uses ray optics to compare the monochromatic X-ray emission produced by a free electron passing through either **a** a crystal with constant interlayer spacings or **b** a crystal with a chirp in the interlayer spacings. In panel (**a**), a collimated X-ray beam is generated, while in panel (**b**), the beam focuses on a point. The illustrations are not to scale

Free-electron-driven coherent X-ray emission incorporates two different mechanisms^56^: parametric X-ray radiation (PXR) and coherent bremsstrahlung (CBS). Radiation by either of these mechanisms has the same dispersion relation, which relates the emission direction to the crystal structures. In a regular periodic crystalline structure shown in Fig. 1a, monochromatic collimated X-ray beams excited by normally incident electrons follow the direction $\cos \varphi = \frac{1}{\beta } - \frac{{n\lambda }}{d}$, where *φ* is the radiation angle relative to the electron velocity, $\beta = v/c$ is the normalized electron velocity, *λ* is the X-ray wavelength, *d* is the interlayer spacing, and *n* is the integer emission order. The above dispersion relation implies that the generated radiation can be made to converge by chirping the interlayer spacing *d*, as sketched in Fig. 1b. The fundamental reason enabling this type of control of the radiation is the relative coherence in the emission from different positions along the electron trajectory. The coherence arises from the fact that the same electron triggers radiation from different layers along its trajectory. Interestingly, our scheme for generating shaped X-rays is similar to (but intrinsically different from) metalenses^57^, which also gradually shifts the phase of the incident coherent light from subwavelength-spaced optical scatterers. Unlike metalenses, which require external light sources, our scheme integrates electron-driven light generation and tailoring in the same process, bypassing the need for subsequent X-ray optical components.

Chirping the interlayer spacing is challenging in the X-ray regime because focusing requires deep-subwavelength manipulation of the interlayer spacing. In this context, vdW materials constitute a versatile platform enabling the required precise adjustment of the interlayer spacings. For example, by intercalating external atoms in vdW materials, the interlayer spacing can be continuously increased by more than a factor of two^31–34^. In addition, vdW heterostructures assembled by materials of similar structure but different chemical compositions can also display quasi-continuous variations of interlayer spacings. For example, WSe_2_ and TaSe_2_ both adopt a hexagonal crystalline structure, but have a 2% difference in interlayer spacings.

Since PXR and CBS share the same dispersion, we concentrate on PXR to design the X-ray focusing effect, while noting that the same conclusions can be applied to CBS. We further note that the contributions from PXR and CBS are comparable in our regime of interest (table-top electron sources)^58^. Since the focusing conditions derived below apply to the polar angle, the resulting X-rays will be focused along a circle that is cylindrically symmetric around the electron trajectory (see Supplementary Section 6). Thus, the focal region forms a 2D belt. The circle of the focus is defined by a point $(x_0,z_0)$ in the $x - z$ plane that satisfies

1 $$\frac{{z_0 - z_i}}{{\sqrt {x_0^2 + \left( {z_0 - z_i} \right)^2} }} = \frac{1}{\beta } - \frac{{n\lambda }}{{d + \delta d\left( {z_i} \right)}}$$

where $d + \delta d(z_i)$ is the interlayer spacing for a layer located at *z*_*i*_. Equation (1) is obtained by combining the collimated X-ray dispersion relation with the spatially modulated interlayer spacing. The interlayer spacing variation $\delta d(z_i)$ implied in the above equation varies almost linearly with the vertical location *z*_*i*_ under the condition $z_i \ll \sqrt {x_0^2 + z_0^2}$. Since the electron is orders of magnitude more energetic than the energy lost in a single inelastic event, the effect of electron energy loss is negligible (see Supplementary Section 7).

**Fig. 2:**
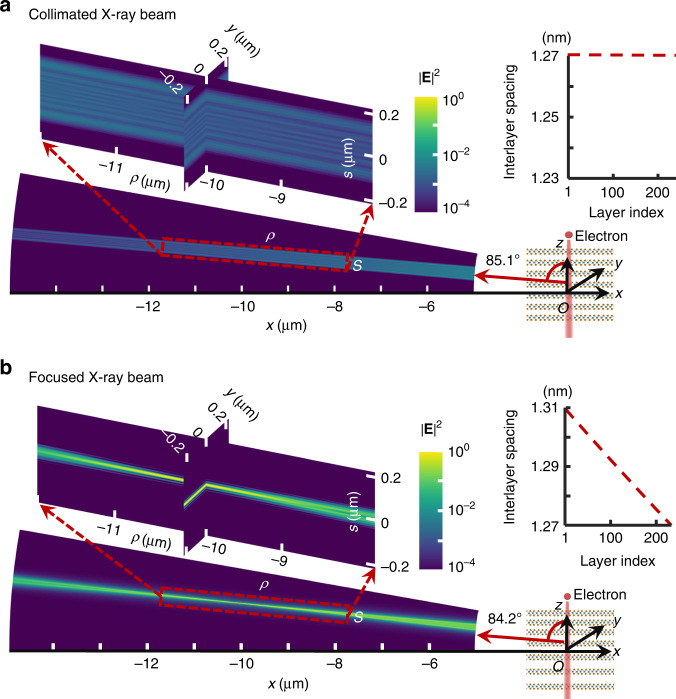
Focused X-ray beam from a vdW heterostructure with chirped interlayer spacings. We compare **b** a focused X-ray beam profile with **a** a collimated one based on the material TaSe_2_. The resulting X-ray beam width is ~10 nm in (**b**) and ~300 nm in (**a**). The inset in the top-right corner of each panel shows the interlayer spacing chirp along the *z* direction. The color scales are the same on both panels, emphasizing the field enhancement at the focused hotspot in (**b**). The *ρ-s* frames are rotated clockwise relative to the *x-z* frame, so that the *ρ* direction points along the emitted beam axis. The enlarged figures highlight the transverse distribution of the beam profiles. The sample thicknesses (300 nm), photon energy (4 keV), and electron kinetic energy (1 MeV) are the same in both panels

We compare in Fig. 2 collimated and focused X-ray beams based on the material TaSe_2_. When a free electron of 1 MeV traverses a regular TaSe_2_ multilayer structure, shown in Fig. 2a, a collimated X-ray beam is emitted (this example corresponds to radiation order *n* = 4). However, when the same electron passes a customized heterostructure with an interlayer spacing chirp (top-right insert of Fig. 2b), the emitted X-ray is focused with a focal length of $f = \sqrt {x_0^2 + z_0^2} = {{{\mathrm{10}}}}\;\mu {{{\mathrm{m}}}}$. Moreover, the beam width of the focused X-ray beam at the focal spot is ~10 nm, much smaller than that of the collimated one (~300 nm). The simulated beam width is consistent with the Abbe diffraction limit, as discussed in Supplementary Section 5. Details on the numerical simulation can be found in the Methods.

**Fig. 3:**
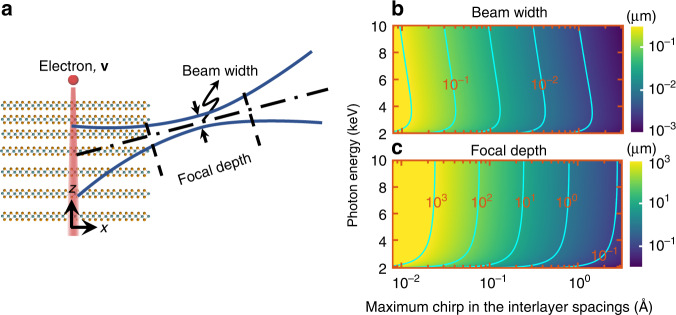
Width and focal depth of focused X-ray beams. **a** Sketch defining the beam width and focal depth. The latter is the distance between the two transverse planes in which the beam width is √2 larger than at the focal spot. **b**, **c** Distributions of beam width and focal depth as a function of chirp in the interlayer spacings and photon energies. The horizontal axis shows the maximum difference between the interlayer spacings at the top and bottom layers of the heterostructure. The contours (cyan curves) are labeled by the respective values. The electron kinematic energy is set to 1 MeV and the minimum interlayer spacing is 12.70 Å

The parameters quantifying the focused hotspot, such as the beam width and focal depth, are tunable as a function of the interlayer spacing chirp and photon energy. These two parameters are directly related to the effective NA (see Methods). We find that, from ray optics considerations, the NA of the focused X-ray beam studied here depends on the maximum chirp in the interlayer spacings rather than on the total sample thickness (see Methods), under the approximation of smooth chirp. Nevertheless, as shown in the discussion, the sample thickness provides a diffraction limit on the hotspot. We plot in Fig. 3 the distribution of beam width and focal depth based on the same layout as in Fig. 2, while varying the photon energy and the maximum chirp in the interlayer spacings (provided a minimum interlayer spacing of 12.70 Å). The distributions of the beam width and NA as functions of sample thickness and interlayer spacing chirp are provided in more detail in Supplementary Section 5.

**Fig. 4:**
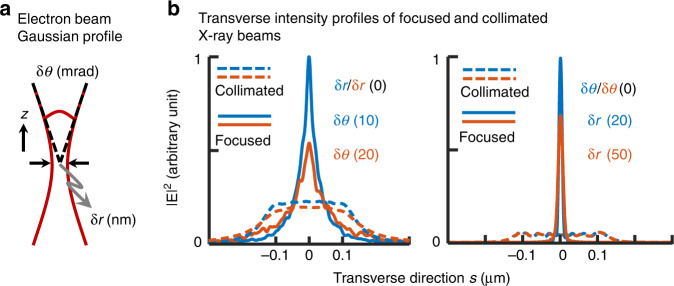
Comparison of the intensity profiles of focused X-ray beams and collimated ones, showing their dependence on the incident e-beam parameters. **a** Gaussian e-beam parameters: root-mean-square (rms) divergence angle δ*θ* and spot size δ*r*. **b** Transverse intensity profiles of the focused and the collimated X-ray beams at the distance *ρ* = 10 μm from the source. In the left column, the e-beam spot size is δ*r* = 0 and the divergence angles are δ*θ* = 10 mrad and 20 mrad. In the right column, the e-beam divergence angle is δ*θ* = 0 and the spot sizes are δ*r* = 20 and 50 nm

The above analysis is based on one free electron traversing the heterostructure and, thus, directly extends to the result of using highly collimated electron beams (e-beams). However, realistic e-beams have a finite divergence angle due to the space charge effect^19^ and electron scattering inside the crystal^56^ (Supplementary Sections 1, 2). The e-beams can usually be modeled by a Gaussian electron density profile, as sketched in Fig. 4a, with a root-mean-squared (rms) divergence angle $\delta \theta$ and an rms spot size $\delta r$. We compare the transverse profiles of focused and collimated X-ray beams at the distance $\rho = {{{\mathrm{1}}}}0\;\mu {{{\mathrm{m}}}}$ based on the layout in Fig. 2b. The results (Fig. 4b) show that the beam width at the focal spot gets broader by increasing the divergence angle and the spot size of the e-beam, but it still remains far superior to the beam-width of the collimated X-ray beam.

**Fig. 5:**
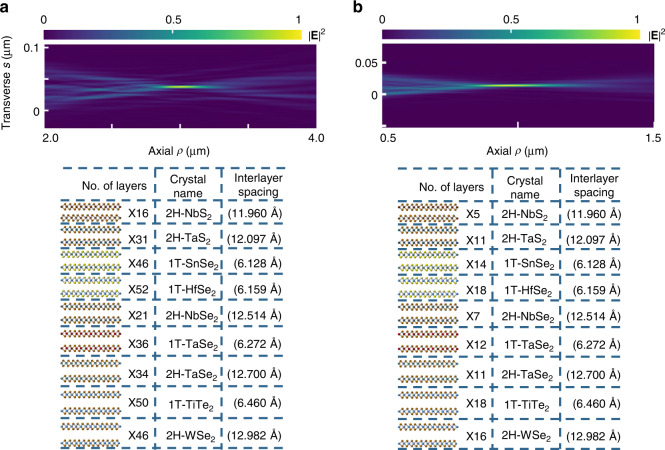
Examples of focused X-ray beams created in multilayer heterostructures comprising vdW materials of similar crystal structure but different compositions. **a**, **b** Two different X-ray focusing schemes with the material configurations listed in the respective tables. The emitted X-rays self-focus along the respective *ρ* axes, which are rotated clockwise by angles of **a** 86.4° and **b** 86.5° relative to the electron trajectory. The observed focal lengths are **a** 3 μm and **b** 1 μm. The sample thickness is **a** 300 nm and **b** 100 nm. 1T and 2H denote two phases of vdW materials with hexagonal crystal structure, in which there are either one (1T) or two (2H) layers in each vertical unit cell. Twice the interlayer spacing of crystals in the 1T phase is counted to compare with the interlayer spacings of crystals in the 2H phase. Both panels share the same photon energy (4 keV) and e-beam kinetic energy (1 MeV)

The chirp in the interlayer spacings of the heterostructure can be realized by assembling different vdW materials of similar crystal structures but different chemical compositions^29,30,42^, as illustrated in Fig. 5. In our numerical calculations, for simplicity, the interlayer spacing at the interface of the two materials is taken as the average value of the adjacent interlayer spacing, although in general it can be tuned to other values by adjusting the relative orientation of adjacent layers^59^. Exemplary designs for focused X-ray beams with focal distances of 3 and 1 μm are shown in Figs. 5a, b, respectively. Both heterostructures are assembled from the same nine different types of vdW materials, but with different configurations to fit two target focal distances. Note that each configuration in Fig. 5 is an approximation to the chirp designated by Eq. (1), such that slight modifications of these configurations would not alter the results.

The flux density at the focal spot (e.g., $\rho = 3\;\mu {{{\mathrm{ m}}}}$ in Fig. 5a) is about 10^11^ photons sec^−1^ mm^−2^ 0.1% BW^−1^ (Supplementary Section 3). The performance is comparable with that of the state-of-the-art X-ray tubes^60^, and yet our scheme is highly monochromatic. We calculate the flux from a Gaussian electron beam of current 10 μA, energy 1 MeV, rms divergence angle 21 mrad, and spot size 19 nm, considering the space charge effect and electron multiple scattering.

## Discussion

### Limitations due to wave optics and quantum effects

**Fig. 6:**
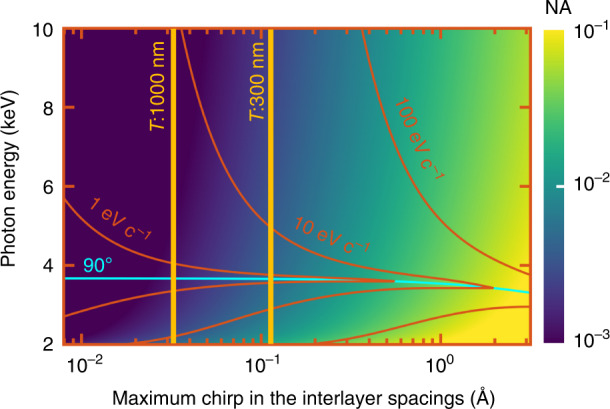
Limits to the obtainable X-ray numerical aperture (NA) due to wave optics and quantum mechanical effects. From wave optics: the focal depth and length are inversely proportional to NA^2^ and NA, respectively. Therefore, there is a lower bound for the NA, because the focal depth should be smaller than the focal length. The two vertical yellow lines delineate the condition of the ratio focal depth⁄focal length = 1 for samples of thicknesses *T* = 1000 and 300 nm. The ratio decreases for larger sample thicknesses and larger chirp in the interlayer spacings. A smaller ratio corresponds to a shorter axial focal region (i.e., a less elongated hotspot). From quantum mechanical considerations: the photon coherence at the focal spot is tied to the electron coherence. The red curves indicate the lower bound for the electron transverse momentum range required for photons to interfere coherently at the focal spot. Generally, a larger electron transverse coherence is needed for achieving a bigger NA. Intriguingly, when the focused X-ray beam is emitted along directions normal to the electron velocity (cyan curve), it requires relatively smaller electron coherence. The calculation is based on the platform of Fig. 2b, with a minimum interlayer spacing of 12.70 Å

The achievable values of beam widths and focal depths are limited by wave optics and quantum effects. From wave optics, the focal spot is spread axially over a length known as the focal depth^61^ $2\lambda /{{{\mathrm{NA}}}}^2$, where *λ* is the X-ray wavelength. For meaningful focusing, the focal depth cannot exceed the focal length $\left( {T\sin \varphi } \right)/\left( {2\;{{{\mathrm{NA}}}}} \right)$, where *T* is the sample thickness, *φ* is the emission angle of the focused X-ray beam, and *T* sin *φ* is the effective dimension of the self-focusing source. The yellow lines in Fig. 6 provide two examples of the lower boundaries of the regions (to the right of the lines) satisfying the above-mentioned limitations of wave optics. Following the noted limitations, microscale to centimeter-scale focal lengths can be achieved by varying the sample thickness, as shown in Supplementary Section 4.

We now move on to investigate the quantum mechanical limitations of free-electron-driven X-ray emission and focusing. Specifically, we explore the circumstances under which the different output photon states are expected to coherently interfere and yield the classically predicted X-ray hotspot. This cannot be taken for granted, since an electron is, in fact, not a point charge but a wave packet of finite size, implying a limited coherent momentum range for the emitted photons. This limit arises from electron recoil during emission^62–66^, which is neglected in the classical picture.

The incident electron recoils while emitting each photon. As a result, the emitted photonic state is entangled with the post-emission electron state. The coherence of the X-ray emission (${{{\mathrm{{\Delta}}}}}{{{\mathbf{k}}}}$) is limited by the combination of uncertainties in the electron coherent momentum ($\hbar {{{\mathrm{{\Delta}}}}}{{{\mathbf{p}}}}$) and in the crystal reciprocal lattice vectors (${{{\mathrm{{\Delta}}}}}{{{\mathbf{g}}}}$). That is, ${{{\mathrm{{\Delta}}}}}{{{\mathbf{p}}}} \,>\, {{{\mathrm{{\Delta}}}}}({{{\mathbf{k}}}} + {{{\mathbf{g}}}})$. Further analysis shows that a gradual change in the crystal reciprocal lattice vectors **g** helps maintain the axially (*z*-direction) photon coherence, that is, ${{{\mathrm{{\Delta}}}}}(k_z + g_z) \approx 0$. In contrast, the transverse (*x*-direction) photon coherence is limited by the transverse momentum uncertainty of the electrons (see Methods). The red curves in Fig. 6 indicate the lower bound for the electron transverse coherent momentum range that enables photon coherent interference for the corresponding NA. Larger transverse coherent momentum ranges correspond to potentially smaller X-ray spots, as predicted by an extended version of the uncertainty principle that here relate the properties of the electrons and the photons. In this respect, experiments have already demonstrated sub-nanometer e-beam spots^67^ (e.g., in scanning transmission electron microscopy), corresponding to hundreds of ${{{\mathrm{eV}}}}\;c^{ - 1}$ transverse coherent momentum.

## Conclusion and outlook

In conclusion, we propose a novel X-ray lensing paradigm based on vdW heterostructures with gradually varying interlayer spacings. Our concept can be realized by relying on state-of-the-art interlayer spacing customization techniques in vdW materials, such as intercalation^32–34^, pressure^35,36^, temperature^37^, optical excitation^38,39^, and vertical assembly of different vdW materials^29,41–44^. When free electrons traverse a heterostructure with suitably customized interlayer spacings, a focused X-ray beam is achieved. The NA of the resulting lensing effect is tunable as a function of the generated photon energy and the extent of interlayer spacing modulation. We compare and discuss various state-of-the-art X-ray lensing paradigms in Supplementary Section 8, in terms of the resulting focal length, beam width, and coherent/incoherent focusing, as well as the footprint of the sources needed.

Furthermore, we apply a quantum mechanical analysis to test our concept and find that for the focused X-ray beam to form necessitates a sufficient transverse electron momentum uncertainty. Without meeting this condition, the electron undergoes recoil associated with the photon emission process and thus becomes entangled with the photon^62–66^, preventing the coherent interference necessary for the formation of the focused beam. The crystal lattice variation helps maintain a high degree of photon coherent interference because part of the recoil associated with the photon emission process is absorbed by the crystal. Therefore, we conclude that only the transverse electron uncertainty affects the interference of the emitted X-ray wave at the focal spot. The important role of electron recoil has only been identified and appreciated recently in free-electron radiation phenomena^68,69^, and so far, only in the optical regime.

Our work paves the way for novel customizable X-ray sources. Breakthroughs in the synthesis and manipulation of vdW materials over the past decade promise great versatility for customized crystalline structures. We envision more methods of shaping X-rays directly at the source with the advent of more complex crystal structures. Going beyond the vertically designed heterostructures that we proposed here, crystal structures can be laterally customized^70,71^. For example, using bilayer moiré patterns can form 2D heterostructures^49^, or applying strain engineering^72–74^ of vdW materials can control crystal structures, such as scrolls^75^, folds^76,77^, bubbles^78–82^, ripples^74,83–85^, buckles^86,87^, crumples^88^, tents^82,89^, and more. These concepts open new avenues that leverage exotic geometrical configurations in the design and control of X-ray emission.

## Methods

### Free-electron-driven X-ray radiation from a 2D crystal layer

The electromagnetic field that accompanies a free electron moving with constant velocity **v** can be written in ${{{\mathbf{r}}}} - \omega$ space as^90^

2 $$\begin{array}{l}{{{\mathbf{E}}}}^{{{{\mathrm{ele}}}}}\left( {{{{\mathbf{r}}}},\omega } \right) = 2\pi i\mu _0e\omega {\int} {\frac{{d^3{{{\mathbf{q}}}}}}{{\left( {2\pi } \right)^3}}\left( {\overline{\overline {{{\mathrm{I}}}}} - \frac{{{{{\mathbf{qq}}}}}}{{k^2}}} \right) \cdot {{{\mathbf{v}}}}\frac{{e^{i{{{\mathbf{q}}}} \cdot \left( {{{{\mathbf{r}}}} - {{{\mathbf{r}}}}_{{{\mathrm{e}}}}} \right)}}}{{k^2 - q^2}}\delta \left( {\omega - {{{\mathbf{q}}}} \cdot {{{\mathbf{v}}}}} \right)} \\ \qquad\qquad\quad= \frac{{ie}}{{\varepsilon _0v_z}}{\int} {\frac{{d^2{{{\mathbf{Q}}}}}}{{\left( {2\pi } \right)^2}}\frac{{k{{{\mathbf{v}}}}/c - {{{\mathbf{q}}}}}}{{k^2 - q^2}}e^{i{{{\mathbf{q}}}} \cdot \left( {{{{\mathbf{r}}}} - {{{\mathbf{r}}}}_{{{\mathrm{e}}}}} \right)}}\\\qquad\qquad\quad = {\int} {\frac{{d^2{{{\mathbf{Q}}}}}}{{\left( {2\pi } \right)^2}}{{{\mathbf{E}}}}^{{{{\mathrm{ele}}}}}\left( {{{{\mathbf{Q}}}},z,\omega } \right)e^{i{{{\mathbf{Q}}}} \cdot {{{\mathbf{R}}}}}} \end{array}$$

where $\omega$ and $k = \omega /c$ are the photon angular frequency and wavenumber in vacuum, respectively; $\mu _0$ and $\varepsilon _0$ the vacuum permeability and permittivity, respectively; $- e$ the electron charge; **Q** the $x - y$ component of the wave vector **q**; the *z*-component of this vector is understood to be ${{{\mathbf{q}}}} \cdot {{{\hat{\mathrm z}}}} = q_z = (\omega - {{{\mathbf{Q}}}} \cdot {{{\mathbf{v}}}})/v_z$ in the last two lines; **R** the $x - y$ component of the position **r**; ${{{\mathbf{r}}}}_{{{\mathrm{e}}}} = ({{{\mathbf{R}}}}_{{{\mathrm{e}}}},0)$ is the point where the free electron traverses the 2D plane at time $t = 0$; $\overline{\overline {{{\mathrm{I}}}}}$ is the unit dyadic; *v*_*z*_ is the *z*-component of velocity **v**; and ${{{\mathbf{E}}}}^{{{{\mathrm{ele}}}}}\left( {{{{\mathbf{Q}}}},z,\omega } \right)$ is the 2D Fourier transform of ${{{\mathbf{E}}}}^{{{{\mathrm{ele}}}}}\left( {{{{\mathbf{r}}}},\omega } \right)$.

We consider a 2D crystal located at the *z* = 0 plane. The 2D crystal is simulated as a dipole array^90^, with bound electrons around each atom being encapsulated in an effective dipole quantified through an associated X-ray atomic polarizability $\alpha (\omega )$^91,92^. Under the assumption of isotropic polarizabilities, the induced dipoles are oriented along the direction of the electron electric field. Here, $\alpha (\omega )$ is derived from the tabulated X-ray scattering factor^93^. The scattering field produced by the dipole array in response to the incident plane wave ${{{\mathbf{E}}}}^{{{{\mathrm{ele}}}}}\left( {{{{\mathbf{Q}}}},z,\omega } \right)$ is^90,94^

3 $$\begin{array}{l}{{{\mathbf{E}}}}^{{{{\mathrm{sca}}}}}\left( {{{{\mathbf{r}}}},\omega ,{{{\mathbf{G}}}}} \right) = \frac{{i\alpha \left( \omega \right)}}{{2A\varepsilon _0}}{\int} {\frac{{d^2{{{\mathbf{Q}}}}}}{{\left( {2\pi } \right)^2}}\frac{{k^2 - {{{\mathbf{kk}}}}}}{{k_z}} \cdot {{{\mathbf{E}}}}^{{{{\mathrm{ele}}}}}\left( {{{{\mathbf{Q}}}} + {{{\mathbf{G}}}},z = 0,\omega } \right)e^{i{{{\mathbf{Q}}}} \cdot {{{\mathbf{R}}}} + {{{\mathrm{i}}}}{{{\mathbf{G}}}} \cdot {{{\mathbf{R}}}}_{{{\mathrm{a}}}} + ik_z\left| z \right|}} \\ \qquad\qquad\qquad\quad= - \frac{{\alpha \left( \omega \right)e}}{{2Av_z\varepsilon _0^2}}{\int} {\frac{{d^2{{{\mathbf{Q}}}}}}{{\left( {2\pi } \right)^2}}\frac{{k^2 - {{{\mathbf{kk}}}}}}{{k_z}} \cdot \frac{{k{{{\mathbf{v}}}}/c - {{{\mathbf{Q}}}} - {{{\mathbf{G}}}} - q_z\hat z}}{{k^2 - \left| {{{{\mathbf{Q}}}} + {{{\mathbf{G}}}}} \right|^2 - q_z^2}}e^{i{{{\mathbf{G}}}} \cdot \left( {{{{\mathbf{R}}}}_{{{\mathrm{a}}}} - {{{\mathbf{R}}}}_{{{\mathrm{e}}}}} \right) + i{{{\mathbf{Q}}}} \cdot \left( {{{{\mathbf{R}}}} - {{{\mathbf{R}}}}_{{{\mathrm{e}}}}} \right) + ik_z\left| z \right|}} \end{array}$$

where *A* is the area of one unit cell, $k_z = \sqrt {k^2 - Q^2}$, **G** is the in-plane ($x - y$ directions) reciprocal lattice vector, and **R**_a_ is the atom position inside one unit cell.

The final scattering field is the coherent sum of the electric fields represented by Eq. (3), arising from different layers for a common **G**. However, the contributions from different **G**s are summed incoherently due to the lack of coherence for different electron impact parameters **R**_e_^63^.

It is noteworthy that the dipole array radiation captures both PXR and transition radiation (TR), the latter being a boundary effect from an electron traversing the interface between two media. The interaction range of TR can be estimated from the formation length equation, which is $L_{{{{\mathrm{TR}}}}} = \frac{{2\gamma ^2c}}{\omega } = 8.6$ Å for a 4 keV photon and 1 MeV electron. Therefore, TR is contributed only by the first and last layers of the heterostructure. TR is negligible compared to PXR, which results from the constructive interference of radiation from each of the many layers. Another typical free electron radiation process in the optical regime, Cherenkov radiation (CR), is not present here because the refractive indices of materials are generally less than unity at X-ray frequencies. Some materials, such as Be and Si, do have refractive indices slightly greater than one in small spectral intervals near the radiation absorption edges, but in such cases, ultra-relativistic electrons—which we do not consider here—would be required to excite any substantial CR in the X-ray regime^95^.

### The basic design and derivation of the numerical aperture (NA)

We consider the focus at $(x_0,z_0)$ (with rotational symmetry along the electron trajectory). The heterostructure is placed between $z = - T/2$ and $z = T/2$, with the interlayer spacings at $z = - T/2$ and $T/2$ being $d + {{{\mathrm{{\Delta}}}}}d$ and *d*, respectively. From Eq. (1), we have

$$\frac{{z_0 - T/2}}{{\sqrt {x_0^2 + \left( {z_0 - T/2} \right)^2} }} \approx \frac{{z_0}}{{\sqrt {x_0^2 + z_0^2} }} - \frac{{x_0^2T}}{{2\left( {x_0^2 + z_0^2} \right)^{\frac{3}{2}}}} = \frac{1}{\beta } - \frac{{n\lambda }}{{d + {\Delta}d}} = {{{\mathrm{A}}}}$$

and

4 $$\frac{{z_0 + T/2}}{{\sqrt {x_0^2 + \left( {z_0 + T/2} \right)^2} }} \approx \frac{{z_0}}{{\sqrt {x_0^2 + z_0^2} }} + \frac{{x_0^2T}}{{2\left( {x_0^2 + z_0^2} \right)^{\frac{3}{2}}}} = \frac{1}{\beta } - \frac{{n\lambda }}{d} = {{{\mathrm{B}}}}$$

where $\beta = v/c$ is the normalized electron velocity, *n* is the radiation order, and *λ* is the X-ray wavelength. In terms of the parameters A and B, introduced to simplify the representation, the focal length reduces to $f = \left[ {1 - \left( {\frac{{{{{\mathrm{A}}}} + {{{\mathrm{B}}}}}}{2}} \right)^2} \right]\frac{T}{{{{{\mathrm{B}}}} - {{{\mathrm{A}}}}}}$. Then, from this equation, the numerical aperture admits the expression

5 $${{{\mathrm{NA}}}} = \frac{{T\sin \theta}}{{2f}} = \frac{{\sin \theta \;\left( {{{{\mathrm{B}}}} - {{{\mathrm{A}}}}} \right)}}{{2\left[ {1 - \left( {\frac{{{{{\mathrm{A}}}} + {{{\mathrm{B}}}}}}{2}} \right)^2} \right]}} = \frac{1}{2}\frac{{{\Delta}dn\lambda}}{{d^2\left[ {1 - \left( {\frac{1}{\beta} - \frac{{n\lambda}}{d}} \right)^2} \right]}}$$

where the emission angle *θ* relative to the *z*-direction follows the condition $\cos \theta = \frac{{{{{\mathrm{A}}}} + {{{\mathrm{B}}}}}}{2}$, and the effective source size normal to the emission direction is *T*sin *θ*.

The NA without the small thickness approximation is discussed in Supplementary Section 5, where we show that the maximum possible NA is limited by the electron transverse coherence.

### Coherent processes in both collimated and focused X-ray beams

We investigate the X-ray emission from a quantum perspective. The initial electron-photon state is described as a superposition of electron momentum states $\left| {{{\mathbf{p}}}} \right\rangle$ by means of $\left| i \right\rangle = \mathop {\sum }\nolimits_{{{\mathbf{p}}}} \frac{1}{{\sqrt V }}\psi \left( {{{\mathbf{p}}}} \right)\left| {{{\mathbf{p}}}} \right\rangle \otimes \left| 0 \right\rangle$, where *V* is the quantization volume, $\left| 0 \right\rangle$ is the photon vacuum state, and $\psi \left( {{{\mathbf{p}}}} \right)$ is the momentum-space wave function (i.e., the amplitude of each electron momentum). Within first-order perturbation theory, the final electron-photon state is described as $\left| f \right\rangle = \mathop {\sum }\nolimits_{{{{\mathbf{p}}}}^\prime ,{{{\mathbf{k}}}}} \frac{1}{{\sqrt V }}\psi \left( {{{{\mathbf{k}}}},{{{\mathbf{p}}}}^\prime } \right)\left| {{{{\mathbf{p}}}}^\prime } \right\rangle \otimes \left| {1_{{{\mathbf{k}}}}} \right\rangle$, where the sum now includes the emitted photon wave vector **k**, $\psi \left( {{{{\mathbf{k}}}},{{{\mathbf{p}}}}^\prime } \right)$ is the corresponding wave function, and $\left| {1_{{{\mathbf{k}}}}} \right\rangle$ is the final one-photon state. The reduced photon-density operator is $\rho _{{{{\mathrm{ph}}}}} = \frac{1}{V}\mathop {\sum }\nolimits_{{{{\mathbf{p}}}}^\prime } \mathop {\sum }\nolimits_{{{{\mathbf{k}}}},{{{\mathbf{k}}}}^\prime } \psi \left( {{{{\mathbf{k}}}},{{{\mathbf{p}}}}^\prime } \right)\psi ^ \ast \left( {{{{\mathbf{k}}}}^\prime ,{{{\mathbf{p}}}}^\prime } \right)\left| {1_{{{\mathbf{k}}}}} \right\rangle \left\langle {1_{{{{\mathbf{k}}}}^\prime }} \right|$. This expression shows that coherent photon states are entangled to the same final electron state. In what follows, we analyze quantum coherent processes and the corresponding requirements for focused X-ray beams.

**Fig. 7:**
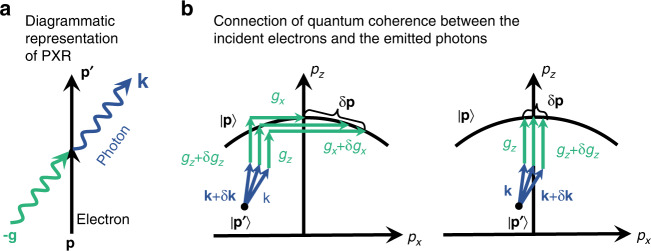
Quantum coherence underlying the formation of focused X-ray beams. In panel **a**, we show the parameters **k**, **p**, **p**′, and **g** are the wave vectors of the emitted photon, the initial and final electron, and the reciprocal lattice vector, respectively. Panel **b** sketches processes associated with electron-driven radiation from a heterostructure with designed interlayer spacings. Each path of arrows denotes a transition satisfying momentum and energy conservation. The initial electron states, which transition to the final electron states |**p**′〉 by emitting fixed-energy photons, are distributed along an isoenergetic energy surface (black curve). Different photon coherent states (blue arrows) are entangled to the same electron final state |**p**′〉. The variation of in-plane reciprocal lattice vector **g** assists in the connection of quantum coherence between the incident electron and the emitted photon. The right column of (**b**) represents emission processes corresponding to a zero in-plane reciprocal lattice vector

A focused X-ray beam requires quantum coherent interference of photon states within a relatively large momentum range. To achieve this, the initial electron states should (I) be distributed along an isoenergetic surface (the black curve in Fig. 7b), and (II) have momentum uncertainties ${{{\mathrm{{\Delta}}}}}{{{\mathbf{p}}}} \,>\, {{{\mathrm{{\Delta}}}}}\left( {{{{\mathbf{g}}}} + {{{\mathbf{k}}}}} \right)$, where **g** and **k** are the reciprocal lattice vectors and photon wave vectors, respectively. These two requirements are constrained by the conservation of momentum and energy for monochromatic photon emission. The isoenergetic curves in Fig. 7 have very small curvature for relativistic electrons, resulting in ${{{\mathrm{{\Delta}}}}}p_x \gg {{{\mathrm{{\Delta}}}}}p_z$ and negligible ${{{\mathrm{{\Delta}}}}}p_z$ near the $p_z$ axis. Therefore, for the normally incident electrons here studied, we only need to consider the transverse electron coherence.

The transverse electron wave vector uncertainties ${{{\mathrm{{\Delta}}}}}p_x$ limit the range of photon coherence via ${{{\mathrm{{\Delta}}}}}p_x \ge {{{\mathrm{{\Delta}}}}}(g_x + k_x)$. Just like in the heterostructure examples examined in Fig. 5, we consider in-plane 2D crystal structures that are nonuniform across the layers, with $\hbar c{{{\mathrm{{\Delta}}}}}g_x = 470\;{{{\mathrm{eV}}}}$ for the first in-plane order. Therefore, to have coherent interference between photons scattered by the first in-plane order across different layers, the electron transverse wave vector uncertainties must satisfy $\hbar c{{{\mathrm{{\Delta}}}}}p_x \ge 470\;{{{\mathrm{eV}}}}$. However, for the specific zero in-plane order (right column of Fig. 7b) and the heterostructures with uniform two-dimensional crystal structures, we have that only the condition ${{{\mathrm{{\Delta}}}}}p_x \ge {{{\mathrm{{\Delta}}}}}k_x$ is required for photon coherent interference. In Fig. 2b, we consider a scenario in which only the interlayer spacing is varied, resulting in $\hbar c{{{\mathrm{{\Delta}}}}}p_x \ge 10\;{{{\mathrm{eV}}}}$, which is readily achievable in electron microscopy^67^.

## Supplementary information

Supplementary Material

## Data Availability

All data needed to evaluate the conclusions in the paper are present in the paper and the Supplementary Material. Additional data related to this paper may be requested from the authors.
