# Supplementary material for: Free-electron interactions with van der Waals heterostructures: a source of focused X-ray radiation
Source: Light Sci Appl. 2023 Jun 16;12:148. doi: 10.1038/s41377-023-01141-2 (PMC10272160; doi:10.1038/s41377-023-01141-2)
Supplement: Supplementary file 1 — Supplementary Material [file 41377_2023_1141_MOESM1_ESM.pdf]

Supplementary Information for
Free-electron interactions with van der Waals heterostructures: a source of focused X-ray radiation

Xihang Shi[1], Yaniv Kurman[1], Michael Shentcis[1],

Liang Jie Wong[2], F. Javier García de Abajo[3,4] and Ido Kaminer[1†]

[1]Solid State Institute and Faculty of Electrical and Computer Engineering, Technion – Israel Institute of Technology, 32000 Haifa, Israel
[2]School of Electrical and Electronic Engineering, Nanyang Technological University, Singapore, Singapore
[3]ICFO–Institut de Ciencies Fotoniques, The Barcelona Institute of Science and Technology, Castelldefels, Spain
[4]ICREA–Institució Catalana de Recerca i Estudis Avançats, Passeig Lluís Companys 23, 08010 Barcelona, Spain
† Corresponding Author: kaminer@technion.ac.il

Contents

Section 1 | Electron beam divergence due to the space charge effect 2

Section 2 | Electron beam divergence by electron scattering 3

Section 3 | The photon flux density at the focal spot 4

Section 4 | The focal lengths of focused X-ray beams 5

Section 5 | Variation of numerical aperture and beam width with sample thicknesses and interlayer spacing chirps 6

Section 6 | The radially symmetric distribution of the focused X-ray beam 8

Section 7 | Interlayer spacing chirp considering electron energy loss in the medium 9

Section 8 | Comparison of state-of-the-art X-ray lensing paradigms 10

References 10

Section 1 | Electron beam divergence due to the space charge effect

The space charge effect, i.e., the inter-electron repulsion, is much more significant for electron beams (e-beams) with larger electron currents and smaller spot sizes. To calculate the induced beam divergence, we model an e-beam with a uniform charge distribution across an elliptical cross-section[1], as shown in the inset of Fig. S1a. The e-beam is moving along the z direction with current I and velocity v. In the electron rest frame $x' - y' - z'$, the electrons are repelled by the electrostatic potential

$$\Phi'(x', y') = -\frac{\rho'}{2\varepsilon_0}\left(\frac{x'^2 Y + y'^2 X}{X + Y}\right) \tag{1}$$

where ρ' is the charge density, ε_0 the vacuum permittivity, $(x' = x, y' = y)$ the position of a point inside the electron beam, X and Y the semi-major axis and the semi-minor axis. The charge density in the rest frame is $\rho' = \rho \cdot \gamma^{-1}$, where γ is the Lorentz factor and $|\rho| = I/(\pi XYv)$.

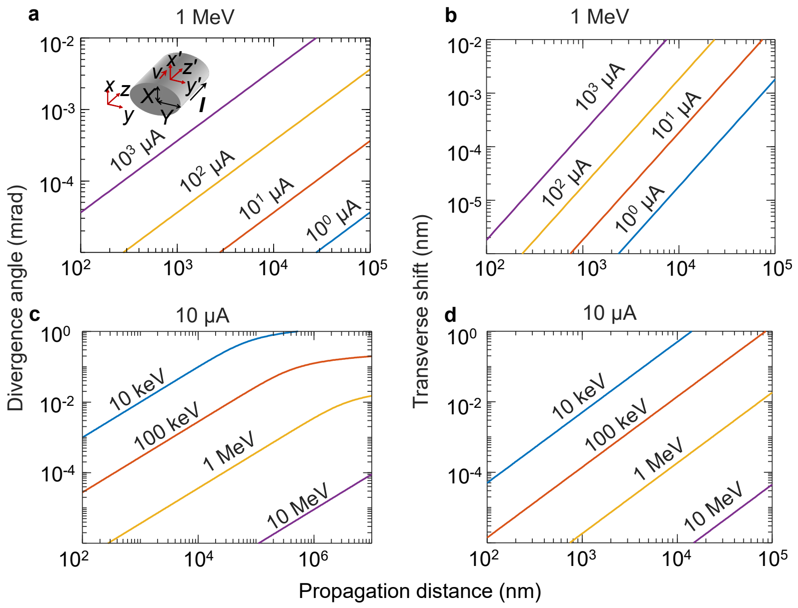

Figure S1 | Divergence of circular electron beams induced by the space charge effect. (**a,b**) The electron energy is set to 1 MeV. (**c,d**) The current is set to 10 μA. The initial beam width at the focal plane is chosen as $2X_0 = 2Y_0 = 30$ nm for all the panels.

The axes of the e-beam evolve in the electron rest frame according to

$$\frac{\mathrm{d}^2 X}{\mathrm{d}t'^2} = \frac{\mathrm{d}^2 Y}{\mathrm{d}t'^2} = -\frac{e\rho'}{m\varepsilon_0}\left(\frac{XY}{X + Y}\right) \tag{2}$$

where $-e$ is the electron charge, m the rest mass of an electron. Using the relation $dt' = dz/(v\gamma)$, where z is the propagation distance of the e-beam in the lab frame, the e-beam axes evolve in the lab frame as

$$\frac{\mathrm{d}^2X}{\mathrm{d}z^2} = \frac{\mathrm{d}^2Y}{\mathrm{d}z^2} = \frac{2C}{X+Y} \tag{3}$$

where $C = -\frac{1}{m\gamma^3v^3}\frac{eI}{2\pi\varepsilon_0}$. The solutions to the above equations are

$$z = \frac{(X_0+Y_0)}{\sqrt{2C}}\frac{\sqrt{\pi}}{2}\mathrm{Erfi}\left[\sqrt{\ln\frac{X+Y}{X_0+Y_0}}\right]$$
$$X = Y + X_0 - Y_0 \tag{4}$$

where $X_0 = X(z=0)$ and $Y_0 = Y(z=0)$ are the e-beam width at the focal plane ($z = 0$). The e-beam divergence angle is

$$\theta(z) = \mathrm{atan}\left(\frac{\mathrm{d}X}{\mathrm{d}z}\right) = \mathrm{atan}\left(\sqrt{2C\ln\frac{X+Y}{X_0+Y_0}}\right) \tag{5}$$

We show in Fig. S1 the divergence angle and transverse shift of an e-beam with different currents and electron energies.

Section 2 | Electron beam divergence by electron scattering

Considering the electron scattering by the crystals, most of the deflection is due to Coulomb scattering from nuclei as described by the Rutherford cross section[2]. The root-mean-square (rms) divergence angle (θ^{rms}) and the transverse shift (T^{rms}) are described by

$$\theta^{\mathrm{rms}}(z) = \sqrt{2}\frac{13.6\ \mathrm{MeV}}{\beta cp}\sqrt{\frac{z}{L_\mathrm{R}}}\left(1 + 0.038\ln\left(\frac{z^2}{L_\mathrm{R}\beta^2}\right)\right)$$
$$T^{\mathrm{rms}}(z) = \frac{1}{\sqrt{3}}z\theta^{\mathrm{rms}} \tag{6}$$

where z is the propagation distance, p the electron momentum, and L_R the radiation length in the medium. We plot in Fig. S2 the e-beam divergence inside the heterostructure with the configuration tabled in Fig. 5a.

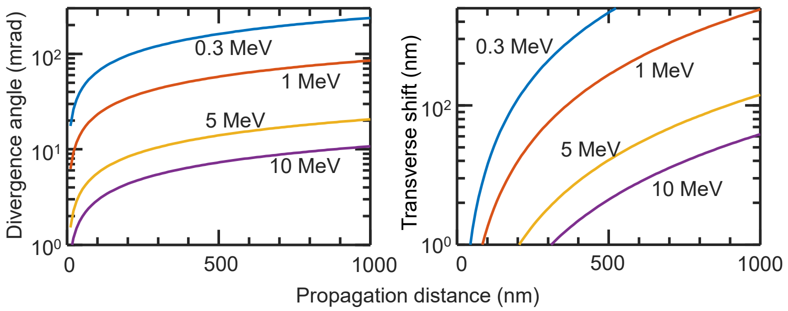

Figure S2 | Divergence of electron beams induced by electron multiple scattering. The electron kinematic energies are 0.3 MeV, 1 MeV, 5 MeV, and 10 MeV.

Combining Fig. S2 with the robustness analysis of the focusing effect from a divergent electron beam in Fig. 4 of the main text, we find that the focusing effect is favored by a more energetic electron, which is more collimated with the same propagation distance. For example, if we use 300 keV electrons, the thickness of the heterostructure should be around 100 nm or less, considering the tradeoff between the electron beam divergence and the focusing effect. Semi-relativistic electrons (60 keV–300 keV) can be found in transmission electron microscopes (TEMs), whereas modestly relativistic electrons (500 keV–5 MeV) are available in room-sized high-voltage transmission electron microscopes (HVTEMs)[3], in which a series of radio-frequency (RF) cavities are used. Highly relativistic electrons (5 MeV-10 MeV) require room-sized linear electron accelerators.

Section 3 | The photon flux density at the focal spot

The power flow traversing an infinitesimal patch $\boldsymbol{\sigma} = \sigma\hat{n}$ (small enough so that the power flow is uniformly distributed in it) is

$$\begin{aligned}\Delta E &= \int_{-\infty}^{\infty} dt\big(\mathbf{E}(\mathbf{r},t)\times\mathbf{H}(\mathbf{r},t)\big)\cdot\boldsymbol{\sigma} \\ &= \int_{0}^{\infty} d\omega\hbar\omega\left[\frac{2\mathrm{Re}\big(\mathbf{E}(\mathbf{r},\omega)\times\mathbf{H}^*(\mathbf{r},\omega)\big)\cdot\hat{n}}{2\pi\hbar\omega}\right]\sigma\end{aligned} \tag{7}$$

where $\left[\frac{2\mathrm{Re}\left(\mathbf{E}(\mathbf{r},\omega)\times\mathbf{H}^*(\mathbf{r},\omega)\right)\cdot\hat{n}}{2\pi\hbar\omega}\right]\sigma$ is the number of photons traversing the patch $\boldsymbol{\sigma}$ per angular frequency per electron. Therefore, the photon flux density is

$$\frac{2\mathrm{Re}\big(\mathbf{E}(\mathbf{r},\omega)\times\mathbf{H}^*(\mathbf{r},\omega)\big)\cdot\hat{n}}{2\pi\hbar\omega}\cdot\frac{I}{e} \tag{8}$$

where I is the current. In Fig. S3, we plot the photon flux density at the focal plane $\rho = 3.0$ μm in Fig. 5a.

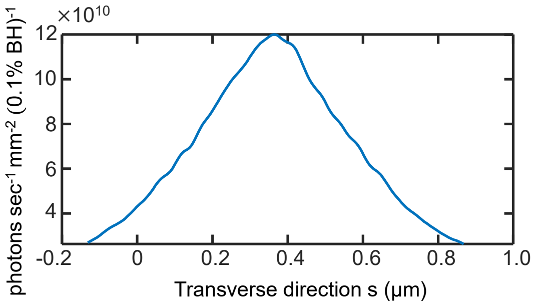

Figure S3 | The flux density at the focal plane of Fig. 5a from a Gaussian electron beam. The e-beam has 10 μA current and 1 MeV kinetic energy. The e-beam has a Gaussian profile as in Fig. 4a, with a rms divergence angle $\delta\theta$ =21 mrad (the average of the rms divergence angle of a 1 MeV electron along a 300 nm trajectory) and a rms spot size δr = 19 nm (the average of the rms spot size of a 1 MeV electron along a 300 nm trajectory).

Section 4 | The focal lengths of focused X-ray beams

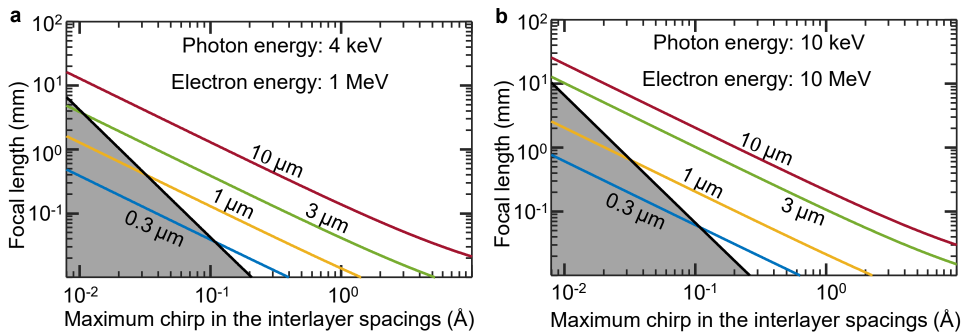

Figure S4 | The focal lengths of focused X-ray beams. a and **b** show the results with different photon energies and electron energies. The shaded regions, which are bounded by the focal depths, denote the regions where the focused hotspots are blurred with the background. The maximum chirp in the interlayer spacings represents the interlayer difference between the bottom layer and the top layer (which is 12.70 Å). Different curves are from different sample thicknesses.

Section 5 | Variation of numerical aperture and beam width with sample thicknesses and interlayer spacing chirps

We plot in Fig. S5 the diffraction-limited numerical aperture (NA) using Eq. (5) of the main text, and the beam width of the focal spot, which is given by the Abbe diffraction limit $\frac{\lambda}{2\,\mathrm{NA}}$. The vertical axis is the variation of the interlayer spacing per 100 nm sample thickness, assuming that the chirp varies smoothly in the interlayer spacings.

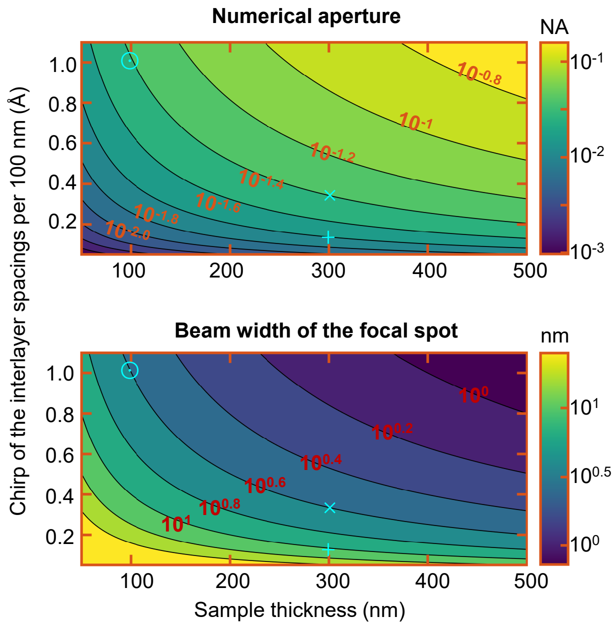

Figure S5 | The diffraction-limited numerical aperture and beam width. The numerical aperture is obtained using Eq. (5) of the main text, and the beam width is the Abbe diffraction limit. The marks +, ×, and ⊙ denote the configurations from Figs. 2a, 5a, and 5b, respectively. The photon energy is 4 keV and the electron energy is 1 MeV. The vertical axis is the variation of the interlayer spacing per 100 nm sample thickness.

The configurations of the heterostructures in Figs. 2 and 5 are marked in Fig. S5. In the setup of Fig. 2, the simulation shows a beam width (full width at $1/e$ of the intensity peak) of ~10 nm, consistent with the diffraction limit in Fig. S5. The setups in Fig. 5 do not have a smooth chirp, but still achieve the diffraction-limited beam width. Both of the beam widths from the numerical simulation are 4.2 nm for Fig. 5a and Fig. 5b, whereas they both have a diffraction-limited beam width of 3.9 nm from Fig. S5 .

In Fig. S6, we estimate the largest achievable NA without making use of the small sample thickness approximation adopted in Eqs. (4) and (5). We see that the NA can reach a value close to one under large and smooth chirp conditions in the heterostructure (e.g., 1 nm chirp per 100 nm within 500 nm interaction length, implying that the chirp of the interlayer spacing should gradually vary up to 5 nm).

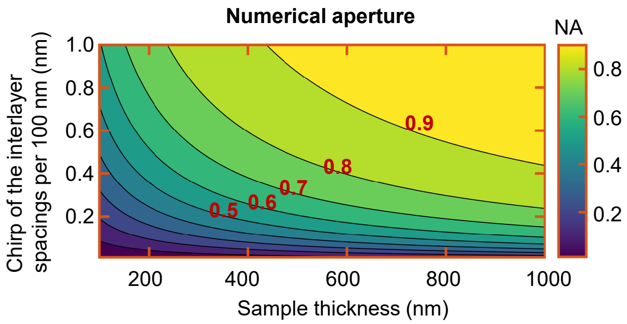

Figure S6 | Numerical aperture without using the small sample thickness approximation. The minimum interlayer spacing is 10 nm, and an ultra-relativistic electron ($\beta \approx 1$) is assumed. The radiation order is $n = 4$, and the photon energy is 4 keV, just as in the main text.

However, the achievable NA is limited by the electron transverse coherence Δp_x. We have shown in Fig. 6 of the main text that the restriction on the electron coherence is relieved when the focused X-ray beam is emitted along directions normal to the electron trajectory. This implies that the focal spot is at the plane $z = 0$, accompanied by $\Delta p_x > \frac{2\pi}{\lambda}(1 - \sqrt{1 - \mathrm{NA}^2})$, where λ is the photon wavelength. Then we have $\mathrm{NA} < \sqrt{1 - \left(1 - \frac{\lambda}{\text{electron focal spot size} \times 4\pi}\right)^2} \sim \sqrt{1 - \left(1 - \frac{0.3\ \text{nm}}{1\ \text{nm} \times 4\pi}\right)^2} \approx 0.2$, where a 4 keV photon and 1 nm electron focal spot are considered. The value increases to $\mathrm{NA} < 0.31$ when the size of the electron focal spot reduces to 0.5 nm.

Finally, we calculate the broadening of the X-ray beam width from realistic e-beams, as illustrated in Fig. 4a, with a finite divergence angle and spot size. A comparison between a diffraction-limited e-beam and a realistic e-beam is shown in Fig. S7.

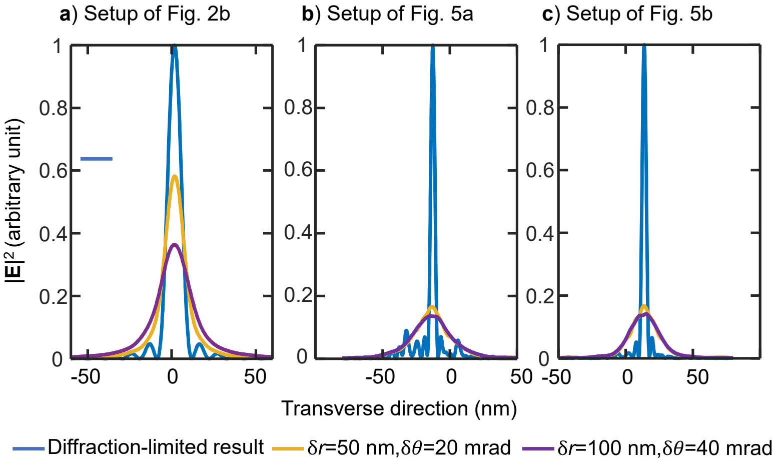

Figure S7 | Beam width of the focused X-ray hotspot from realistic divergent electron beams. Gaussian e-beam parameters, such as the root-mean-square (rms) divergence angle $\delta\theta$ and spot size δr, are illustrated in Fig. 4a.

Section 6 | The radially symmetric distribution of the focused X-ray beam

The X-ray focus is not a two-dimensional spot as in a regular lens. The focusing of the X-ray beam forms a radially symmetric circle surrounding the electron beam, as shown in Fig. S8.

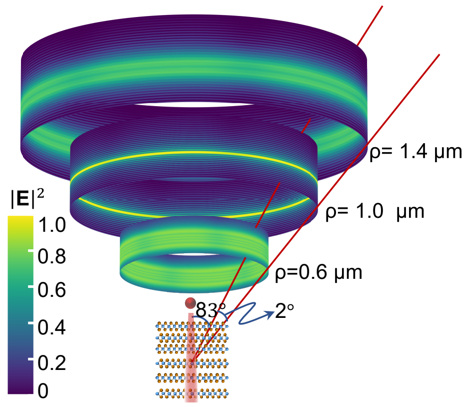

Figure S8 | Radially symmetrical distribution of the focused X-ray beam. The presented simulation is based on the setup of Fig. 2b. Considering the average over electron normal trajectories relative to the crystal structure, we obtain a nearly radially symmetrical distribution of the focused X-ray beam. Taking into account the radial resolution of the focused hotspot, the focal region is a 2D plane. The angles are not scaled to show the full view of the image.

Section 7 | Interlayer spacing chirp considering electron energy loss in the medium

The electron experiences energy losses from inelastic scattering inside the medium. The stopping power for 1 MeV electrons in a material such as $TaSe_2$ is 1.182 MeV cm^2 g^{-1}, as found in NIST database[4]. Therefore, the electron energy loss upon passing through one layer of $TaSe_2$ is δE=1.182 MeV cm^2 g^{-1} ×8.68 g cm^{-3}×1.27 nm=1.303 eV, where 8.68 g cm^{-3} is the density of $TaSe_2$, and 1.27 nm is the interlayer distance of regular $TaSe_2$.

In order to incorporate the effect of inelastic energy losses in the heterostructure design, we need to replace the constant β in Eq. (1) by $\beta_i = \left(1 - i\frac{\delta E}{E}\right)\beta$, where i represents the i-th layer traversed by the electron. As a result, Eq. (1) from the main text is transformed into

$$\frac{z_0 - z_i}{\sqrt{x_0^2 + (z_0 - z_i)^2}} = \frac{1}{\left(1 - i\frac{\delta E}{E}\right)\beta} - \frac{n\lambda}{d + \delta d(z_i)} \tag{9}$$

where a total energy loss much smaller than the electron energy is assumed. Figure S9 compares the interlayer spacing predicted by Eq. (1) in the main text and Eq. (9) here. We see that the interlayer spacing would have to be corrected by at most 0.34% due to the energy loss experienced by the electrons. This implies that our predicted peak frequency at the focal spot would shift by less than 0.34% in our numerical results.

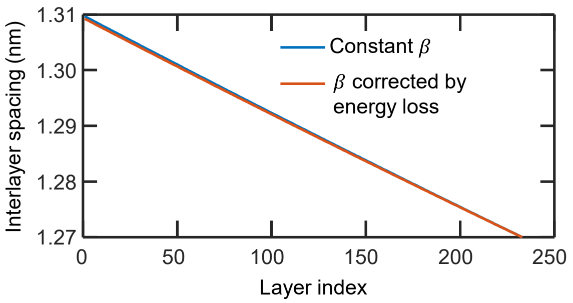

Figure S9 | Interlayer spacing corrected by electron energy loss. We use the setup in Fig. 2b to compare the effect with/without considering electron energy loss. We see that the interlayer spacing corrected by electron energy loss decreases by 0.34% at one end of the heterostructure.

Section 8 | Comparison of state-of-the-art X-ray lensing paradigms

In Table S1, we compare the performance of different X-ray lensing paradigms. The X-ray optics exploiting the coherent X-rays show a good performance in terms of focal length and spot size. However, the required X-ray beam qualities can only be achieved in cumbersome facilities, hindering potentially widespread applications. The compact scheme consisting in combining a polycapillary lens with an X-ray tube suffers from large spot sizes due to incoherent focusing. The parameters of our vdW heterostructure lens listed in the table represent typical results discussed in the main text, incorporating the electron beam divergence inside the crystal. Large focal lengths can also be achieved from ultra-relativistic electrons, as shown in Fig. S4.

Table S1| State-of-the-art X-ray lensing paradigms

	focal length	spot size	limited to large facilities?	coherent focusing?	representative reference
reflection mirror	75 mm	7 nm	Yes	Yes	Nat. Phys. 6, 122–125 (2010)
zone-plate (diffraction)	2.6 mm	16 nm	Yes	Yes	APL 92, 221114 (2008)
refractive lens	1.6 mm	~5 nm	Yes	Yes	PRL 94**,** 054802 (2005)
tapered waveguide	4.9 mm	~ 10 nm	Yes	Yes	PRL 91, 204801 (2003)
polycapillary lens with an x-ray tube	~3 mm	11-14 µm	No	No	Lindqvist, M. Thesis, Uppsala University, 2017
our vdW heterostructure lens	1-10 µm	10-100 nm	No	Yes	this work

References

1. Sacherer, F. J. RMS envelope equations with space charge. *IEEE Transactions on Nuclear Science* **18**, 1105-1107 (1971).
2. Particle Data Group. *et al*. Review of particle physics. *Progress of Theoretical and Experimental Physics* **2020**, 083C01 (2020).
3. Sannomiya, T. *et al*. Transmission electron microscope using a linear accelerator. *Physical Review Letters* **123**, 150801 (2019).
4. Berger, M. J. *et al.* Stopping-Power & Range Tables for Electrons, Protons, and Helium Ions. *NIST Stand Ref Database* **124**, (2017).
